# Recent development of risk-prediction models for incident hypertension: An updated systematic review

**DOI:** 10.1371/journal.pone.0187240

**Published:** 2017-10-30

**Authors:** Dongdong Sun, Jielin Liu, Lei Xiao, Ya Liu, Zuoguang Wang, Chuang Li, Yongxin Jin, Qiong Zhao, Shaojun Wen

**Affiliations:** 1 Department of Hypertension Research, Beijing Anzhen Hospital, Capital Medical University and Beijing Institute of Heart Lung and Blood Vessel Diseases, Beijing, China; 2 Beijing Lab for Cardiovascular Precision Medicine(PXM2017_014226_000037), Beijing, China; 3 Program Director & Medical Officer, Lung Cell and Vascular Biology Program, National Heart, Lung, and Blood Institute, Bethesda, Maryland, United States of America; 4 Department of Medicine, Division of Cardiology, Virginia Commonwealth University School of Medicine, Inova Campus, Falls Church, Virginia, United States of America; The University of Tokyo, JAPAN

## Abstract

**Background:**

Hypertension is a leading global health threat and a major cardiovascular disease. Since clinical interventions are effective in delaying the disease progression from prehypertension to hypertension, diagnostic prediction models to identify patient populations at high risk for hypertension are imperative.

**Methods:**

Both PubMed and Embase databases were searched for eligible reports of either prediction models or risk scores of hypertension. The study data were collected, including risk factors, statistic methods, characteristics of study design and participants, performance measurement, etc.

**Results:**

From the searched literature, 26 studies reporting 48 prediction models were selected. Among them, 20 reports studied the established models using traditional risk factors, such as body mass index (BMI), age, smoking, blood pressure (BP) level, parental history of hypertension, and biochemical factors, whereas 6 reports used genetic risk score (GRS) as the prediction factor. AUC ranged from 0.64 to 0.97, and C-statistic ranged from 60% to 90%.

**Conclusions:**

The traditional models are still the predominant risk prediction models for hypertension, but recently, more models have begun to incorporate genetic factors as part of their model predictors. However, these genetic predictors need to be well selected. The current reported models have acceptable to good discrimination and calibration ability, but whether the models can be applied in clinical practice still needs more validation and adjustment.

## Introduction

The number of people living with hypertension is predicted to be 1.56 billion worldwide by the year 2025[1]. In addition, hypertension contributes to ~13% of the total mortality worldwide[2] and ~7% of the total disability-adjusted life years, creating a tremendous financial burden for both patients and the health-care system[2]. The association between hypertension and traditional risk factors such as age, body mass index (BMI), blood pressure (BP), smoking and family history have been well studied, whereas the roles of genetic variants associated with the incidence of hypertension are less clearly defined[3,4].

In 2013, Echouffo-Tcheugui JB *et al*. published a systematic review of 11 articles with 15 models[5]. Most of these models were carried out in Caucasian populations, and the prediction factors used in these studies were almost identical. Noticeably, none of the above models took genetic factors into consideration, whereas in recent years, more study designs of hypertension risk prediction models have tended not only to have larger patient enrollment size with diverse ethnic backgrounds but also to include genetic factors in these models. Therefore, we conducted this systematic review to summarize the current development status and performance of hypertension prediction models, which would provide updates for health-care providers and policy-makers in the field of hypertension research and clinical practice. This review could also help improve hypertension awareness, identify populations at high risk for hypertension, and determine those individuals who could benefit from early interventions.

## Method

### Search strategy

The research strategy, study selection and analysis methods used in this study followed the guidelines from the Preferred Reporting Items for Systematic Reviews and Meta-Analysis (PRISMA) Statement[6] (S1 Table). We conducted a complete literature search in both PubMed and Embase to retrieve all published reports about hypertension prediction models using the keywords “hypertension”, “high blood pressure”, “prediction model”, and “risk score”. The search strategy was (((prediction model[Title/Abstract]) OR risk score)) AND ((hypertension[Title/Abstract]) OR high blood pressure[Title/Abstract]). The last search was conducted on September 5, 2016. The related references from those retrieved reports were also searched manually to identify any additional published reports. For those identified articles that were not available online, we contacted the authors directly to request copies.

### Inclusion and exclusion criteria

All the retrieved reports were screened independently for inclusion by two researchers from this study. The titles and abstracts of retrieved papers were used as the primary review content for inclusion verification. However, if questioned or unclear, the full article was reviewed prior to inclusion decision. The study’s inclusion criteria include: 1. Reporting a risk assessment tool, e.g., an equation or a risk score system; 2. Predicting the risk incidence of essential hypertension; 3. Published in English-language journals; 4. Conducted in subjects 18 years old or older; 5. Reporting quantitative measures of model performance (preferred but not necessarily required). Exclusion criteria include: 1. Studies only describe association between risk factors and incident hypertension; 2. Simulation studies; 3. Studies predict gestation-related hypertension; 4. Unpublished research data.

### Data extraction and synthesis

Any discrepancy of the independently collected data from the two researchers was resolved by group discussion among all participating project investigators. The following data were extracted from each study: study design, subject characteristics, number of subjects in derivation and validation cohorts, number of subjects who developed hypertension, number of candidate variables considered, variables included in the final model and statistical method used for development of the model. We extracted the area under the curve (AUC) of the receiver operating characteristic or C-statistic to assess the discrimination ability of each model. We also collected the value of Hosmer–Lemeshow χ^2^, and the *p* value of the corresponding test statistic, to assess model calibration ability. Due to the wide spread of differences in risk factors, population, study design, and sufficiency of data, it was impossible to perform meta-analysis in our current study. Instead, we opted to conduct a narrative synthesis of the evidence. However, to provide a nice summary graph, we applied the random effects model meta-analysis to combine the estimates of the AUC from studies with enough data and assessed the between-study heterogeneity, with the use of the Stata statistical software version 12.0(http://www.stata.com/). The data used in meta-analysis was transformed in the way of double arcsine transformations to addresses the problems of confidence limits and variance instability. The potential publication bias was assessed with funnel plot, as well as Begg's and Egger’s test. A P value <.05 indicated significant publication bias.

## Results

**Fig 1:**
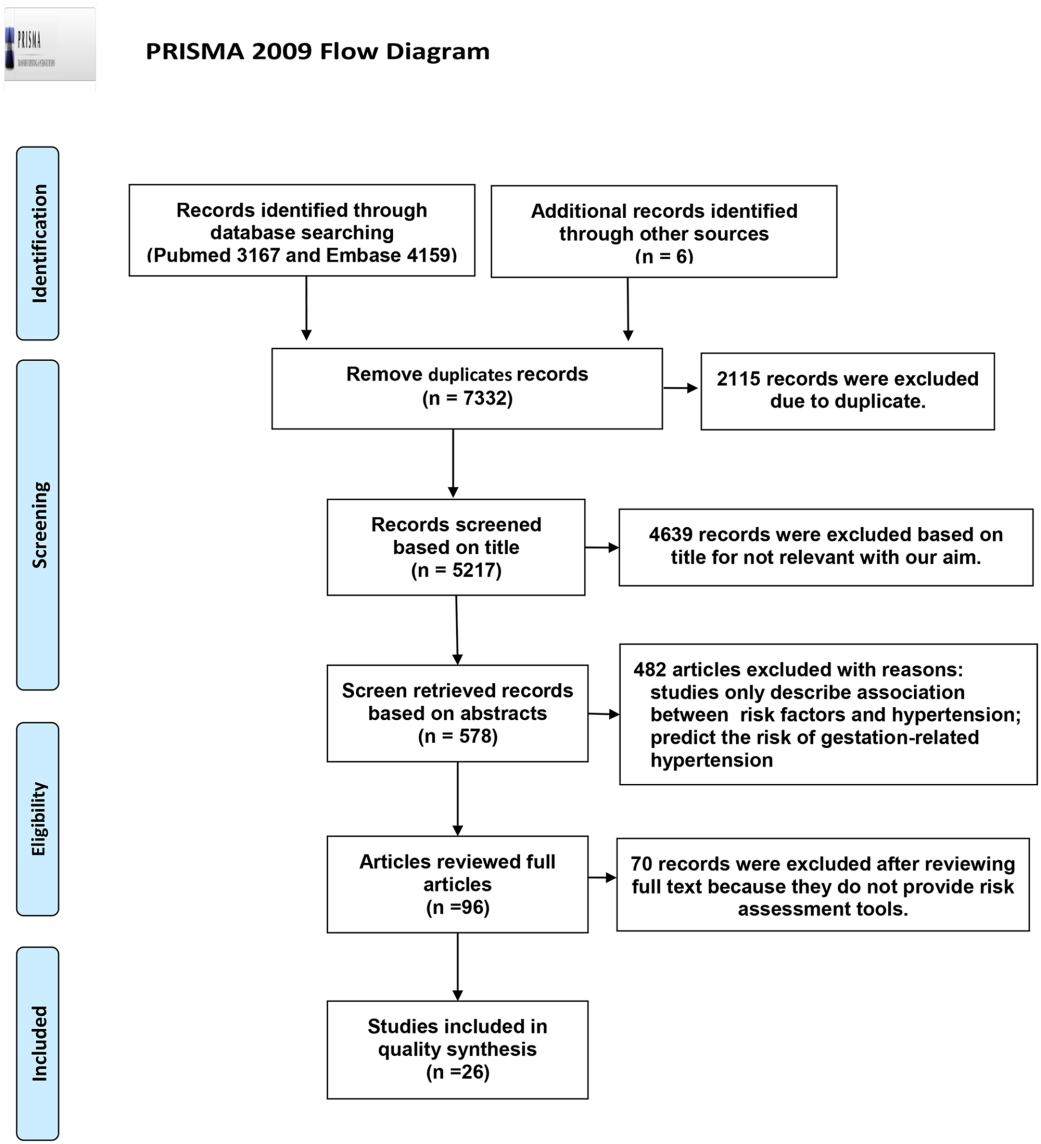
The process of article search and selection. *From*: Moher D, Liberati A, Tetzlaff J, Altman DG, The PRISMA Group (2009). *P*referred *R*eporting *I*terns for *S*ystematic Reviews and *M*eta-*A*nalyses: The PRISMA Statement. PLoS Med 6(6): e1000097. doi:10.1371/joumal.pmed1000097. **For more information, visit** www.prisma-statement.org**.**

The process of the literature search and paper selection, according to PRISMA guidelines, is presented in Fig 1. Our initial literature search resulted in 7332 citations; only 26 articles were selected, reporting 48 prediction models. Table 1 shows the characteristics of these 26 studies, of which 5 were conducted in the US[7–11], 5 in Europe[12–16], 7 in China[17–23], 4 in Korea[24–27], 2 in Japan[28,29], 2 in Iran[30,31], and 1 in India[32]. Among them, only 1 study was carried out in women alone[9]. A total of 162,358 subjects were enrolled in these studies. In the longitudinal studies, participants were followed up for 3 to 30 years. The definition of hypertension among these studies was consistent. Twenty-four studies defined hypertension as either systolic blood pressure (SBP) ≥140 mmHg and/or diastolic blood pressure (DBP) ≥ 90 mmHg, or the use of antihypertensive drugs. Two studies[17,31] defined isolated systolic hypertension as SBP ≥140 mmHg and DBP ≤ 90 mmHg, and isolated diastolic hypertension as DBP ≥ 90 mmHg and SBP ≤ 140 mmHg. Twenty studies used traditional factors only, and 6 studies[11,14,16,19,21,26] also included Genetic Risk Score (GRS) factors (indeed, 2 studies[19,26] used genetic risk factors exclusively). The common predictors included in most models were age, gender, BMI, SBP, DBP, and parental history of hypertension. The SNPs that were used for setting up the GRS system were nearly all derived from the genome-wide association study (GWAS). The number of SNPs used in these studies ranged from 2 to 32 (S2 Table). The AUC or C-statistic of models[11,21] including GRS were superior compared to those without GRS (C-index change = 0.3%–0.5%; *p*<0.05). Twelve studies proposed to build models with logistic regression, 7 with COX regression, 6 with Weibull regression, and 1 with linear regression.

**Table 1:** Characteristics of included articles.

First author	Year	Country/Ethnicity	Study design	Outcomes/total	Age	Definition of hypertension	Follow up (years)	Type of statistic
Pearson	1990	USA/Mixed, mainly Whites	Prospective	104/1130	25 or less	Self-reported use of BP lowering medications ^c^	30	Cox regression analysis
Chih-Jung Yeh	2001	China/Taiwan	prospective	87/2373	≥20	SBP≥ 140 mmHg and DBP< 90 mmHg ^b^^c^	3.23	Cox regression analysis
Nisha I. Parikh	2008	American/whites	prospective	796/1717	20 to 69	JNC—VII definition ^b^^d^	4	Weibull regression model
Nina P. Paynter	2009	American/mainly whites	prospective	derivation 1935/9427; validation 1068/5395	45 and older, females only	Self-report or SBP≥140 mmHg or DBP≥90 mmHg^a^^c^	8	Logistic regression
Mika Kivimäki	2009	England/mainly whites	prospective	1258/8207	35 to 68	JNC—VII definition ^b^	5	Weibull regerssion
Mika Kivimäki	2010	England/mainly whites	prospective cohort	derivation 614/4135; validation 438/2785	35 to 68	JNC—VII definition ^b^	5	Weibull regression
Abhijit V. Kshirsagar	2010	American/whites	prospective	3795/11407	45 to 64	JNC—VII definition ^c^	9	multiple logistic regression
Mohammadreza Bozorgmanesh	2011	Iran/Asians	prospective	805/4656	42	the average of two DBP measurements≥90 mmHg or the average of two SBP ≥140 mmHg or taking antihypertension medication ^b^^c^	6	Weibull proportional hazard regression models
K-L Chien	2011	China/Taiwan	prospective	1029/2506	≥35	JNC—VII definition ^b^	6.15	multivariate Weibull model
Cristiano Fava	2013	Sweden/whites	prospective	NR/10781	NR	JNC—VII definition ^b^	23	Multiple linear and logistic regression
Nam-Kyoo Lim	2013	Korean/Asians	prospective	819/4747	40 to 69	JNC—VII definition ^b^^c^	4	Weibull regression analysis
Henry	2013	Northeast Germany/whites	prospective	training set 166/803; validation set 157/802	20–79	SBP/DBP≥140/90 mmHg ^b^	5	Bayesian networks
Li Guoqi	2014	China/Asians	prospective	1776/3899	35–64	nr	15	logistic regression
Yun-Hee Choi	2014	Mexican Americans	prospective	nr/443	nr	JNC—VII definition	nr	generalized estimating equations method
Yue Qi	2014	China/Asians	case control	1009 with hypertension; 756 normotensive controls	case cohort 64.48±8.53; control 64.23±10.13	JNC—VII definition ^a^	nr	logistic regression
Bum Ju Lee	2014	Korea/Asians	cross-sectional	12789	21–85	SBP/DBP≥140/90 mmHg or physician-diagnosed hypertension	nr	correlation-based feature selection
Nam-Kyoo Lim	2015	Korean/Asians	prospective	nr/5632	40 to 69 years	JNC—VII definition ^b^^c^	4	logistic regression
Toshiaki Otsuka	2015	Japan/Asians	prospective	1633/15025	38.8±8.9	JNC—VII definition ^b^	4	Cox proportional hazards model
Xiangfeng Lu	2015	China/Asians	prospective	2559/7724	35 to 74	JNC—VII definition ^a^^c^	7.9	logistic regression
Wenchao Zhang	2015	China/Asians	prospective	3793/17471	18 to 88	JNC—VII definition ^b^^c^	5	Cox proportional hazards regression model
Minoru Yamakado	2015	Japan/Asians	prospective	424/2637	55.2	JNC—VII definition ^d^	4	logistic regression analysis
Joung-Won Lee	2015	Korea/Asians	prospective	2128 men and 2326 women	40–69	JNC—VII definition ^a^^d^	4	Cox proportional hazard model
Samaneh Asgari	2015	Tehran/Asians	prospective	235/4574	≥20	SBP≥140 mmHg and DBP<90 mmHg ^b^^d^	9.57	Cox proportional hazard regression
Samaneh Asgari	2015	Tehran/Asians	prospective	470/4809	≥20	SBP<140 mmHg and DBP ≥90 mmHg ^b^^d^	9.62	Cox proportional hazard regression
Thirunavukkarasu Sathish	2016	India/blacks	prospective	70/297	15–64	JNC—VII definition ^b^	7.1	logistic regression model
Teemu J. Niiranen	2016	Finland/whites	prospective	nr/2045	≥30	JNC—VII definition ^b^^c^	11	Multiple linear and logistic regression
Chen, Y.	2016	China/Chinese	prospective	2785/12497	40.84±11.34	JNC—VII definition ^b^^c^	4	multivariable backward Cox analyses

Study design is prospective study or cross-sectional study; Outcomes/total means the number of incident hypertension and the total number of participants of each study; Age is expressed as the mean value or range; BP is blood pressure, SBP means systolic blood pressure and DBP means diastolic blood pressure; JNC—VII definition means the definition of hypertension is based on the Joint National Committee (JNC)—VII definition of hypertension (i.e., SBP/DBP ≥140/90 mmHg or use of antihypertension medications).

^a^ means one-time BP measurement was used to define hypertension;

^b^ for average of multiple BP measurements;

^c^ means patient reported anti-hypertensive drugs;

^d^ for abstracted from chart;

First author and year represent study.

### Performance of prediction models

The performance of prediction models is shown in Table 2. AUC ranged from 0.64 to 0.97, and C-statistic ranged from 60% to 90%. The results of pooling 35 models in meta-analysis(S1 File) show the value of AUC as 0.767, 95%CI(0.742, 0.792). The calibration was assessed by Hosmer–Lemeshow χ^2^, suggesting that these models had good calibration ability.

**Table 2:** Characteristics of prediction models.

First author	Year	Model name	Candidate variables (n)	Variables include	AUC/C-statistic	Calibration	Method of validation
Pearson	1990	Johns Hopkins	NR	Age, SBP at baseline, paternal history of hypertension and BMI	NR	NR	NR
Chih-Jung Yeh	2001	ISH risk prediction model	NR	age, DM, and fibrinogen concentration in men, and age and APTT (activated partial thromboplastin time) in women	NR	NR	NR
Chih-Jung Yeh	2001	IDH risk prediction model	NR	elevated BMI, glucose concentration, and uric acid concentration were significant factors in men; BMI was the only significant factor in women.	NR	NR	NR
Nisha I. Parikh	2008	Framingham risk score	11	age, sex, SBP, DBP, BMI, parental hypertension, and cigarette smoking	NR/0.788,95% CI(0.733, 0.803)	Hosmer–Lemeshow χ^2^ = 4.35	NR
Nina P. Paynter	2009	WHS inclusive risk prediction	14	age, BP, BMI, total grain intake, apolipoprotein B, ethnicity, lipoprotein(a), C-reactive protein	NR/0.705	Hosmer–Lemeshow χ^2^ = 2.9(P = 0.94)	Internal validation, split-sample 2:1
Nina P. Paynter	2009	WHS Simplified Model with Lipids	23	Age, BMI, SBP, DBP, ethnicity (Black or Hispanic) and total to HDL- cholesterol ratio	NR/0.705	Hosmer-Lemeshow χ^2^ = 9.4(P = 0.31)	Internal validation, split-sample 2:1
Nina P. Paynter	2009	WHS Simplified Model	23	Age, BMI, race/ethnicity, SBP, and DBP	NR/0.703	Hosmer–Lemeshow χ^2^ = 6.0(P = 0.64)	Internal validation, split-sample 2:1
Mika Kivimäki	2009	Whitehall II risk score	NR	Age, sex, SBP, DBP, BMI, parental hypertension and cigarette smoking	NR/0.80	Hosmer–Lemeshow χ^2^ = 11.5(<20)	Internal validation, split-sample (6:4)
Mika Kivimäki	2010	Whitehall II Repeat measures risk score	NR	repeat measures of BP, weight and height, current cigarette smoking and parental history of hypertension	NR/0.799	predicted-to-observed ratio 0.98, 95% CI(0.89, 1.08). Hosmer–Lemeshow χ^2^ = 6.5	Internal validation, split-sample
Mika Kivimäki	2010	the average blood pressure risk score	NR	average BP, weight and height, current cigarette smoking and parental history of hypertension	NR/0.794	predicted-to-observed ratio 0.96, 95%CI (0.88, 1.06)	Internal validation, split-sample
Mika Kivimäki	2010	the ‘usual’ blood pressure risk score	NR	the ‘usual’ BP, weight and height, cigarette smoking and parental history of hypertension	NR	NR	Internal validation, split-sample
Abhijit V. Kshirsagar	2010	ARIC/CHC risk score	11	Age, level of SBP or DBP, smoking, family history of hypertension, diabetes mellitus, high BMI, female sex, and lack of exercise	0.739 (3years), 0.755 (6 years), 0.800 (9 years) and 0.782 (ever)/nr	NR	Internal validation, split-sample
Mohammadreza Bozorgmanesh	2011	TLGS risk multivariable models	NR	for women: age, waist circumference, DBP, SBP, and family history of premature CVD; for men: age, DBP, SBP, and smoking; for both: the interaction terms between age and SBP, Increasing levels of SBP	NR/0.731 (95% CI 0.706–0.755) for women; 0.741 (95% CI 0.719–0.763) for men	women (Hosmer–Lemeshow χ^2^ = 7.8, P = 0.554) and men (Hosmer–Lemeshow χ^2^ = 8.8, P = 0.452).	NR
Mohammadreza Bozorgmanesh	2011	TLGS risk score	NR	Waist circumference, DBP, family history of premature cardiovascular disease, daily smoking, SBP	NR/0.727 (95% CI 0.709–0.744)	NR	NR
K-L Chien	2011	Taiwan BP clinical risk model	NR	gender, age, BMI, SBP and DBP	0.732,95% CI (0.712,0.752)/NR	Hosmer–Lemeshow χ^2^ = 8.3, p = 0.40	NR
K-L Chien	2011	Taiwan BP clinical risk model	NR	gender, age, BMI, SBP and DBP, white blood count, fasting glucose and uric acid	0.735,95% CI (0.715–0.755)/NR	Hosmer–Lemeshow χ^2^ = 13.2, p = 0.11	NR
Cristiano Fava	2013	Swedish nongenetic risk model	NR	age, sex, age^2^, sex times age, heart rate, obesity, diabetes, hypertriglyceridemia, prehypertension, family history of hypertension, sedentary in spare time, problematic alcohol behavior, married or living as a couple, high-level non-manual work, smoking	NR/0.662	NR	NR
Cristiano Fava	2013	Swedish genetic risk model	29	29 SNPs	NR	NR	NR
Cristiano Fava	2013	Swedish risk model 2	NR	age, sex, age^2^, sex times age, heart rate, obesity, diabetes, hypertriglyceridemia, prehypertension, family history of hypertension, sedentary in spare time, problematic alcohol behavior, married or living as a couple, high-level non-manual work, smoking, 29 SNPs	NR/0.664	NR	NR
Nam-Kyoo Lim	2013	KoGES risk score	NR	age, sex, smoking, SBP, DBP, parental hypertension, BMI	0.79,95% CI (0.764,0.815) /NR	χ^2^ = 13.42, P = 0.0981	NR
Henry	2013	SHIP risk model	42	age, mean arterial pressure, rs16998073, serum glucose and urinary albumin concentrations, interaction between age and serum glucose, interaction between rs16998073 and urinary albumin concentrations	training set 0.78 95% CI(0.74,0.82); validation set 0.79,95%CI (0.75,0.83)/NR	Hosmer–Lemeshow χ^2^ = 11.82 (P = 0.16) for training set; 11.65 (P = 0.17) for the validation set	Internal (1:1) and external validation
Yue Qi	2014	northeastern Han Chinese genetic risk score	10	9 SNPs	NR	NR	NR
Bum Ju Lee	2014	Demographic indices risk prediction model1 for women	41	Height, Age, NeckC, AxillaryC, RibC, WaistC, PelvicC, Rib_Hip, Waist_Hip, Pelvic_Hip, Rib_Pelvic, Axillary_Rib, Chest_Rib, Axillary_Chest, Forehead_Neck	0.696 for Bayes-correlation-based feature selection;0.713 for logistic regression-correlation-based feature selection/NR	NR	NR
Bum Ju Lee	2014	Demographic indices risk prediction model2 for women	41	Height, Age, ForeheadC, NeckC, HipC, Axillary_Hip, Axillary_Pelvic, Chest_Pelvic, Chest_Rib	0.713/NR	NR	NR
Bum Ju Lee	2014	Demographic indices risk prediction model3 for women	41	Height, Weight, BMI, Age, ChestC, Forehead_Hip, Waist_Hip, Chest_Pelvic, Waist_Pelvic, Axillary_Waist, Forehead_Rib, Neck_Axillary	0.721/NR	NR	NR
Bum Ju Lee	2014	Demographic indices risk prediction model 1 for men	41	Age, ForeheadC, NeckC, AxillaryC, ChestC, RibC, WaistC, PelvicC, HipC, Rib_Hip, Waist_Hip, Rib_Pelvic, Waist_Pelvic, Chest_Waist, Forehead_Rib, Chest_Rib, Axillary_Chest, Forehead_Neck	0.64 for Bayes-correlation-based feature selection and 0.637 for logistic regression-correlation-based feature selection/nr	NR	NR
Bum Ju Lee	2014	Demographic indices risk prediction model 2 for men	41	Height, Age, ForeheadC, NeckC, AxillaryC, HipC, Rib_Hip, Pelvic_Hip, Neck_Pelvic, Waist_Pelvic, Chest_Waist, Chest_Rib, Neck_Chest, Axillary_Chest, Forehead_Neck	0.646/NR	NR	NR
Bum Ju Lee	2014	Demographic indices risk prediction model 3 for women	41	Height, ForeheadC, NeckC, AxillaryC, RibC, PelvicC, Forehead_Hip, Chest_Hip, Rib_Hip, Pelvic_Hip, Forehead_Waist, Axillary_Waist, Rib_Waist, Neck_Rib, Axillary_Rib, Chest_Rib, Forehead_Axillary, Forehead_Neck, WHtR	0.652/NR	NR	NR
Li Guoqi	2014	China risk prediction model 1	NR	age, SBP, DBP, BMI and the history of parental hypertension	NR/0.7168	Hosmer-Lemeshow χ^2^ = 3.75	NR
Li Guoqi	2014	China risk prediction model 2	NR	Age, SBP, DBP, BMI and the history of parental hypertension, TG, HDL-C	NR/0.7208	Hosmer-Lemeshow χ^2^ = 3.10	NR
Li Guoqi	2014	China risk prediction score	NR	Age, SBP, DBP, BMI and the history of parental hypertension	NR	NR	NR
Yun-Hee Choi	2014	marginal model	NR	Intercept, Age, Gender, Smoke, Age×gender, Rs10510257 (AA), Rs10510257 (AG), Rs1047115 (GT)	0.839/NR	NR	NR
Yun-Hee Choi	2014	conditional model	NR	Intercept, Age, Gender, Smoke, Age×gender, Rs10510257 (AA), Rs10510257 (AG), Rs1047115 (GT)	0.973/NR	NR	NR
Xiangfeng Lu	2015	InterASIA risk prediction	NR	Model1: Age, sex, and BMI; Model2: Model 1+smoking, drinking, pulse rate, and education; Model3: Model2 + SBP and DBP	NR/Model1:0.650 (0.637–0.663); Model2:0.683 (0.670–0.695);Model3:0.774 (0.763–0.785)	NR	NR
Wenchao Zhang	2015	biomarker-based risk-prediction model	11	inflammatory factor, blood viscidity factor, insulin resistance factor, blood pressure factor, and lipid resistance factor	75.5% for men and 80.1% for women/nr	NR	NR
Nam-Kyoo Lim	2015	Korean genetic risk score	4	rs995322, rs17249754, rs1378942, rs12945290	NR	NR	internal validation fivefold cross-validation
Minoru Yamakado	2015	the PFAA index	19	PFAA index 1, Leucine, Alanine, Tyrosine, asparagine, tryptophan, and Glycine; PFAA index 2, Isoleucine, Alanine, Tyrosine, phenylalanine, methionine and histidine	NR	NR	NR
Toshiaki Otsuka	2015	Japanese risk prediction model	NR	age, BMI, SBP and DBP, current smoking status, excessive alcohol intake, parental history of hypertension	NR/0.861, 95% CI(0.844, 0.877)	Hosmer–Lemeshow χ^2^ = 15.2 P = 0.085 in validation cohort	internal validation Split-sample (80% vs.20%)
Toshiaki Otsuka	2015	Japanese risk score sheet	NR	age, BMI, SBP and DBP, current smoking status, excessive alcohol intake and parental history of hypertension	NR/0.858, 95% CI(0.840,0.876)	Hosmer–Lemeshow χ^2^ = 9.3 P = 0.41 in validation cohort	internal validation Split-sample (80% vs.20%)
Joung-Won Lee	2015	Anthropometric indices risk prediction	NR	BMI; WaistC; waist-to-hip ratio; waist-to-height ratio	NR	NR	NR
Samaneh Asgari	2015	TLGS risk prediction for ISH	17	Age, SBP, BMI, 2 hours post-challenge plasma glucose	NR/0.91	NR	NR
Samaneh Asgari	2015	TLGS risk prediction for IDH	17	Age, DBP, waist circumference, marital status, gender, HDL-C	NR/0.76	NR	NR
Thirunavukkarasu Sathish	2016	rural India risk score	11	age, sex, years of schooling, daily intake of fruits or vegetables, current smoking, alcohol use, BP, prehypertension, central obesity, history of high blood glucose	0.802, 95% CI(0.748–0.856)/NR	Hosmer-Lemeshow P = 0.940	NR
Teemu J. Niiranen	2016	genetic risk prediction model1	32	32 SNPs	NR	NR	NR
Teemu J. Niiranen	2016	genetic risk prediction model2	32	model 1 + age + sex	NR	NR	NR
Teemu J. Niiranen	2016	genetic risk prediction model3	32	model 2 + smoking, diabetes, education, hypercholesterolemia, exercise and BMI	NR/0.803	NR	NR
Chen, Y.	2016	Prediction for men	20	Age, BMI, SBP, DBP, gamma-glutamyl transferase, fasting blood glucose, Drinking, Age by BMI, Age by DBP	0.761, 95% CI(0.752–0.771)	NR	NR
Chen, Y.	2016	Prediction for women	20	Age, BMI, SBP, DBP, fasting blood glucose, total cholesterol, neutrophil granulocyte, Drinking, Smoking	0.753, 95% CI(0.741–0.765)	NR	NR

NR means not reported; BP is blood pressure, SBP is systolic blood pressure and DBP is diastolic blood pressure; BMI is body mass index; AUC means the area under the receiver operating characteristic curve; CI means confidence interval; SNP is single nucleotide polymorphism; NeckC is Neck circumference; AxillaryC: Axillary circumference; RibC: Rib circumference; WaistC: Waist circumference; PelvicC: Pelvic circumference; Rib_Hip: Rib-to-pelvic circumference ratio; Waist_Hip: Waist-to-hip circumference ratio; Pelvic_Hip: Pelvic-to-hip circumference ratio; Rib_Pelvic: Rib-to-pelvic circumference ratio; Axillary_Rib: Axillary-to-rib circumference ratio; Chest_Rib: Chest-to-rib circumference ratio; Axillary_Chest: Axillary-to-chest circumference ratio; Forehead_Neck: Forehead-to-neck circumference ratio; WHtR: Waist-to-height circumference ratio.

### Validation of prediction models

The prediction models of 7 studies[9,10,12,13,15,26,28] were validated in internal cohorts through split samples, with C-statistics ranging from 0.79 to 0.9. Three models were externally validated. The SHIP risk model[15] from northeast Germany was validated by data from the Danish INTER99, comprising 2887 participants, and it performed well, with an AUC of 0.77 (P = 0.74) and the Hosmer–Lemeshow χ^2^ test of 40.6 (P = 2×10^−6^). The KoGES risk score from Korea was externally validated by a large nationwide Korean cohort[33]. The discrimination (AUC = 0.733) and calibration (Hosmer–Lemeshow χ^2^ = 14.85, P = 0.062) of this model were both good. The Framingham model was externally validated by 7 studies[12,15,24,33–36] from different countries (S3 Table).

### Meta-analysis

**Fig 2:**
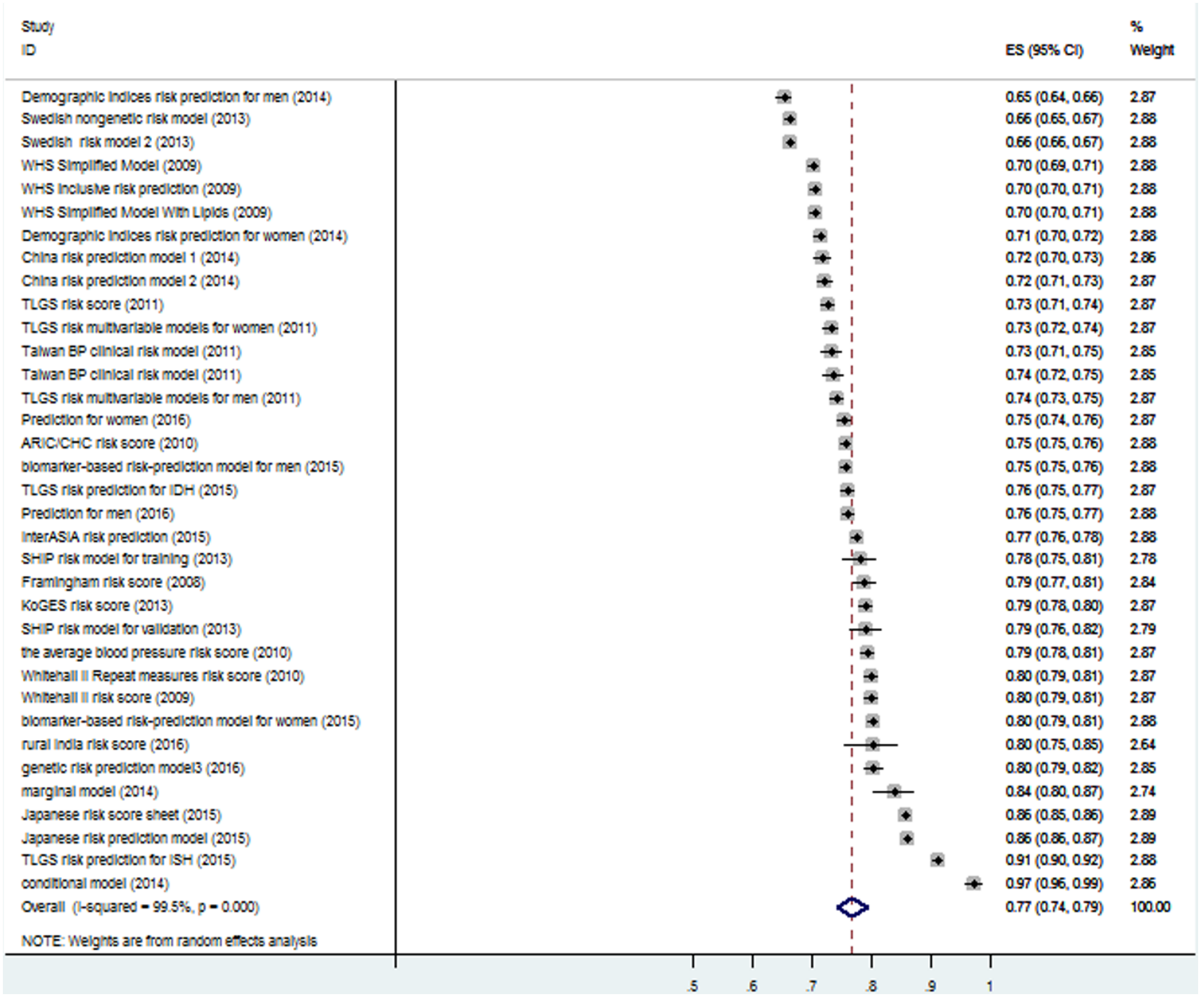
Forest plots of pooling 35 models.

**Fig 3:**
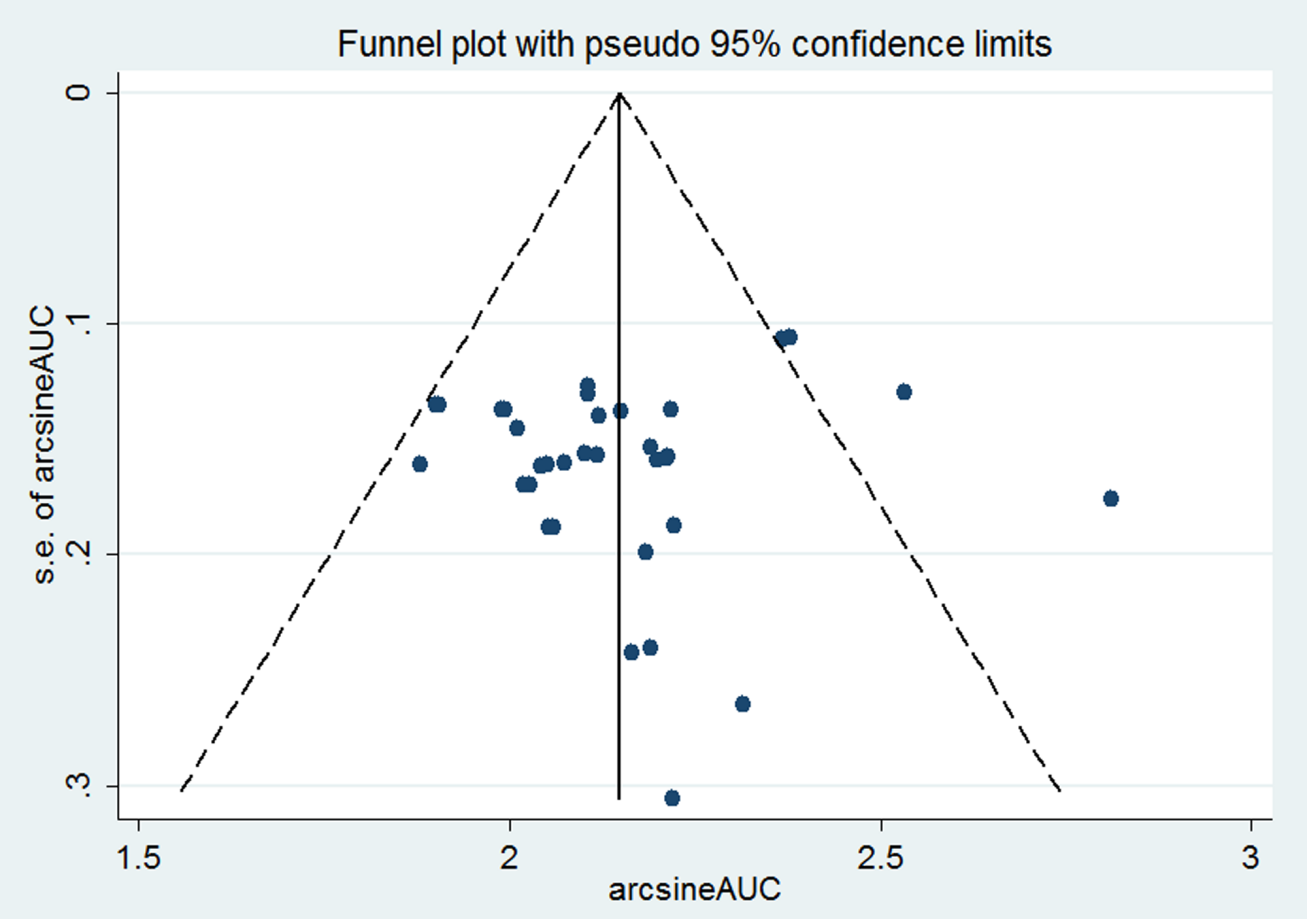
Funnel plot of publication bias.

Results from pooling 35 models in the meta-analysis showed that the AUC was 0.767, 95% CI(0.742, 0.792) indicating the performance of prediction models was well. Fig 2 shows the forest plots of analysis. As expected, the heterogeneity between studies(I-squared = 99.5%, Estimate of between-study variance Tau-squared = 0.0055) was significant(S1 file). Publication bias was evaluated with Funnel plot (Fig 3). The results(P>0.05) indicated no significant publication bias.

## Discussion

This systematic review summarizes the current evidence regarding risk models developed to predict incident hypertension. The prediction models could help identify individuals who are more susceptible to hypertension and prioritize the underlying risk factors that lead to the incidence of hypertension. In addition, it could also help individuals with high risk for hypertension and health-care providers to take preventive interventions earlier.

### Population of studies

Most of these models were derived from American, European or East Asian populations; only one study was carried out in India, and the other 2 were in Iran. It is perceivable that systematic underestimation or overestimation of risk may occur when applying a model constructed from one particular cohort to a distinct ethnic population with different characteristics (the selection of predictors and the genetic background). We found that most prediction models were established in developed countries, and only a few were established in developing or undeveloped countries. Thus, it is imperative to establish reliable predictive models in those countries or regions to help reduce the incidence of hypertension and cardiovascular events caused by high blood pressure.

### Predictors included

The most commonly used predictors include age, BMI, SBP, DBP, etc., which are easy to obtain in clinical practice. A few studies also take blood biochemistry factors or anthropometric parameters[25,27] as predictors (Table 2), which are also part of the routine lab test results in a general physical examination. The biochemistry factors used as predictors include blood glucose, triglycerides, high-density lipoprotein cholesterol (HDL-C), and fibrinogen. It has been reported that the level of blood glucose is associated with high blood pressure[37]. Triglyceride, cholesterol and HDL-C are also known to contribute to blood hyperviscosity[38] and vascular sclerosis, which could lead to the rise of the BP. Since hypertension is also considered as a metabolic disease, the changes of blood biochemical factors could provide important and valuable information for the accuracy of certain hypertension prediction models.

It is well known that the interaction between environmental and genetic factors contributes to the development of hypertension. Theoretically, the prediction models should contain both environmental and genetic predictors. Most of the SNPs used to construct GRS were from GWAS (S2 Table). In the Finnish study[16], results showed that GRS were significantly associated with BP but weakly associated with BP increase and incident hypertension; in contrast, in Hispanic Americans[11], GRS was constructed by 2 SNPs on chromosome 3 alone, and when GRS was added into the model, the improvement of predicting capability measured by the AUC was minor. In a Korean population[26], GRS was constructed by 4 SNPs based on GWAS, which was independently associated with the risk of incident hypertension. Among the 4 SNPs, rs17249754 was the same predictor as that selected in 2 Chinese genetic studies[19,21], and rs1378942 was the same as that used in the Swedish genetic study[14]. However, adding GRS into models with traditional risk factors did not significantly improve the discrimination ability. In the Swedish study[14], when adding cGRS (derived from a simple, unweighted count method) into the traditional model, AUC was marginally, but not significantly, improved (from 0.662 to 0.664). In the 29 SNPs that constructed cGRS, one (rs1378942) was the same as that selected in Korean study[26] and two (rs16998073, rs11191548) were the same as those selected in 2 Chinese studies[19,21]. A couple of factors may contribute to these unfavorable observations. First, since hypertension is a known multigene disease, a limited number of SNPs as representative predictors may not fully reflect the overall contribution and weight of all genetic variants. Second, it is possible that some of those included SNPs were selected without fully considering their potential interactions with other genetic variants or environmental factors.

In contrast, in a Chinese study[21], adding GRS constructed by 22 carefully selected SNPs to the traditional predictors produced an ideal result, as the C-index value improved significantly (C-index change = 0.3%–0.5%; all *p* < 0.05). Among the 22 uncorrelated (*r*^2^ < 0.5) SNPs, 10 were associated with SBP or DBP from published GWAS data obtained from an East Asian population, and 19 SNPs had been identified and verified in a Chinese population. These results clearly suggested that the contribution and value of GRS to a hypertension prediction model heavily depends on the selection of SNPs. Since hypertension is a disease of polygenic inheritance, the selection of SNPs used for GRS construction is thus critical. Using GWAS results as the only source for SNP selection is inadequate, as the characteristics of SNPs obtained from one particular GWAS may not necessarily be suitable for other ethnic populations. More appropriate SNP selection should come from the genetic research results in the same ethnic group. Other SNP selection considerations for GRS construction should include a sufficient number of SNPs, causal relationship between the select genes and disease development, gene-gene or gene-environment interactions, and proper statistical methods to include or exclude gene loci.

At the present stage, genetic markers for predicting hypertension can be of great interest for researchers and basic scientists (and possibly for drug companies), but may not hold much interest for patients. Once genetic factors are included in prediction models, patients cannot use the model for self-assessment, clinicians could face problems explaining the model, and cost for genetic tests can be high. These problems may be resolved with the development of gene-function and gene-sequencing research.

### Model validation

Seven studies validated their prediction models using internal validation. All studies indicated good discriminatory ability and calibration, suggesting that the models could be applied in the original population with satisfactory performance. A Framingham prediction model was validated in external populations by 7 studies (S3 Table). It performed well in a study of African-American and Caucasians in the US[35], a German study[15] and a British study[12]. In a large nationwide Korean cohort,[33]the AUC was acceptable, but this model underestimated hypertension incidence (*p*<0.001) in Korea. In the Multi-Ethnic Study of Atherosclerosis (MESA)[34], including Caucasian, African-American, Hispanic, and Asian (primarily of Chinese descent) participants, the Framingham model showed better discrimination ability than SBP alone or age-specific DBP categories. However, the difference between the observed and the predicted hypertension risks (Hosmer-Lemeshow goodness of fit *p*<0.001) in the MESA study was significant. In contrast, the discrimination (C-statistics = 0.5 to 0.6) and calibration ability (*p*<0.0001) in rural Chinese was poor[36], whereas poor agreement (χ^2^ = 29.73, *p* = 0.0002) underestimated the risk of hypertension in Koreans[24]. The distinct performance in different populations was partially attributed to the various levels of risk predictors and inherited variables. These differences suggested that a model derived from one particular population could not be directly applied to a distinct population, and the fittest model for one particular population is that derived from the same population.

### Heterogeneity

The meta-analysis showed the heterogeneity was significant. The included variables, study designs, number of participants, populations, statistical methods, and follow-up times were different from each other, which might be the source of heterogeneity. We attribute this to the specialization of prediction models, which need to be built for various populations, because no one model could be applied to all people.

### Clinical implications

Currently, a large issue regarding hypertension prediction models/scores is that nobody uses these scores in daily life or clinical practice beyond research publications. Some people even question whether hypertension needs to be predicted, as it can be easily measured with noninvasive, cheap, and accessible methods. The function of these models is not only to predict the occurrence of hypertension but, more meaningfully, to remind patients and physicians to pay attention to BP. What is more, it has been proved that the process of progression from normotensive or prehypertension to hypertension can be delayed or prevented by proper and timely clinical interventions. It is urgent and meaningful for people to conduct timely interventions. The importance of prediction models/scores needs to be widely disseminated by authorities or the media to promote their application.

### Strengths and limitations of existing models

Most of the current predictors are data commonly collected in routine clinical practice, which are relatively easier for both health-care providers and patients to access. Some models are in the form of risk scores, which may still have room to improve but are also convenient to use in routine clinical practice. Furthermore, several models took GRS into account, which could contribute significantly to their prediction accuracy of hypertension. Since the performance of all these prediction models was accepted as good, the application of these models in clinical practice is very promising.

In contrast, several limitations of these prediction models are also noted. First, since not all these studies were specifically designed or conducted for generating prediction models, the clinical data collection may not be complete, and quality of data collected to inform these models also varies greatly; thus, prediction accuracy is a concern. Second, the enrollment number of participants was low in some studies and may not represent the true characteristics of the general population. Third, the various levels of risk predictors and inherited variables between populations made the models inapplicable for other general people. Fourth, a justified method in selecting the suitable SNPs is lacking. Fifth, since most of the BP data were obtained in hospital or clinic settings, the “white coat effect” may influence the outcome of the BP measurement. Sixthly, none of these models have been shown to improve outcomes in prospective research. Lastly, only a few models were indeed validated by internal cohorts, and only 3 were validated in external cohorts. The validation in internal cohort is more or less considered as a repeat of the original cohort and thus may be overoptimistic in its prediction performance results.

## Conclusion

Recently, more and more hypertension prediction models have been reported in different countries and among various ethnic populations. Most of the reported predictors are commonly used in routine clinical practice, and the role of genetic factors is earning more attention. However, the incorporation of genetic variation does not improve the performance significantly for all models. The selection of gene loci is critical, and a justified method in selecting the suitable SNPs is needed. The current reported models have satisfying discrimination and calibration ability, but the validation of these models is still insufficient, which is a critical and required step prior to their broad application in daily clinical practice.

### Perspective of future research

It is obvious that the current prediction models might not be perfect, but they do provide a solid foundation for future studies. Of course, more studies on prediction models of hypertension should be conducted with large enrollment numbers, complete data collection, experienced or well-trained investigators, and appropriate statistical analysis. With the development of genetic research, more hypertension-associated SNPs will be found, and a standard protocol in gene loci selection as a candidate prediction factor will be needed. Indeed, before any models are used as guidelines, they need to be validated in various cohorts and adjusted accordingly.

## Supporting information

S1 Fig Begg’s and Egger’s publication bias plot. (TIF)

S1 Table PRISMA 2009 checklist. (DOC)

S2 Table SNPs of GRS. SNP: single nucleotide polymorphism; GWAS: Genome Wide Association Study; NR: not reported. Rs1378942 was chosen in both Sweden and Korean studies; rs17249754 in Korean and 2 Chinese studies; rs11191548 and rs16998073 from Sweden were the same in two Chinese studies; in two Chinese studies, 7 SNPs (rs17030613, rs16849225, rs1173766, rs11066280, rs35444, rs880315 and rs17249754) were the same.
(DOCX)

S3 Table External validation of the Framingham model. AUC means the area under the receiver operating characteristic curve; CI means confidence interval; JNC—VII definition means the definition of hypertension is based on the Joint National Committee (JNC)—VII definition of hypertension (i.e., SBP/DBP ≥140/90 mmHg or use of antihypertension medications); NR means not reported. First author and year represent study.
(DOCX)

S1 File Results of meta-analysis and publication bias test. (DOCX)
