# Supplementary figures and images for: Recent development of risk-prediction models for incident hypertension: An updated systematic review
Source: PLoS One. 2017 Oct 30;12(10):e0187240. doi: 10.1371/journal.pone.0187240 (PMC5662179; doi:10.1371/journal.pone.0187240)

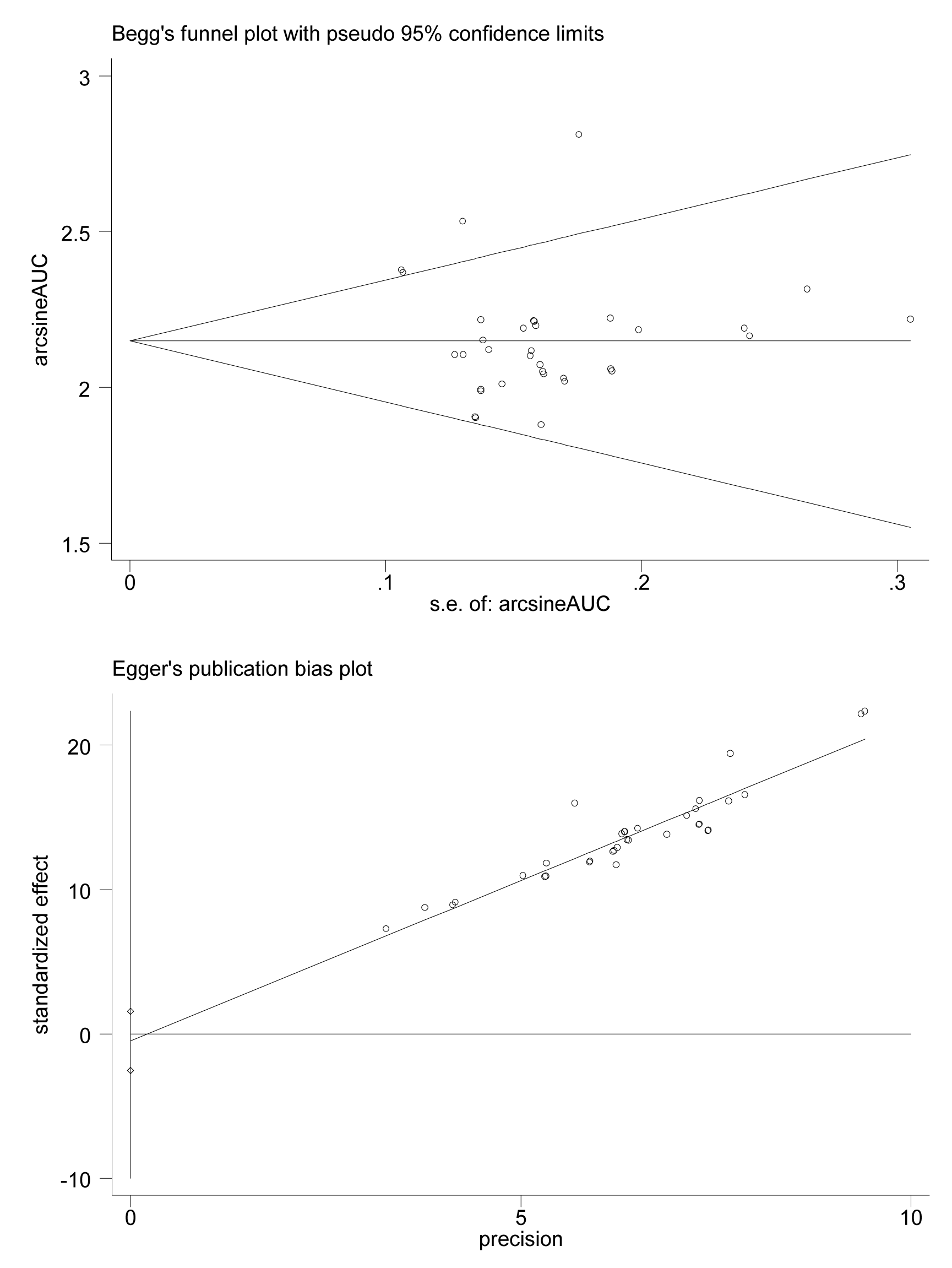

Supplement: S1 Fig — (TIF) [file pone.0187240.s001.tif]
