# Supplementary material for: Recent development of risk-prediction models for incident hypertension: An updated systematic review
Source: PLoS One. 2017 Oct 30;12(10):e0187240. doi: 10.1371/journal.pone.0187240 (PMC5662179; doi:10.1371/journal.pone.0187240)
Supplement: S2 Table — SNP: single nucleotide polymorphism; GWAS: Genome Wide Association Study; NR: not reported. Rs1378942 was chosen in both Sweden and Korean studies; rs17249754 in Korean and 2 Chinese studies; rs11191548 and rs16998073 from Sweden were the same in two Chinese studies; in two Chinese studies, 7 SNPs (rs17030613, rs16849225, rs1173766, rs11066280, rs35444, rs880315 and rs17249754) were the same. (DOCX) [file pone.0187240.s003.docx]

| First author | Year | Country/ethnicity | Number | Source | SNPs |
| --- | --- | --- | --- | --- | --- |
| Cristiano Fava | 2013 | Sweden/whites | 29 | Mainly GWAS | rs17367504, rs633185, rs6015450, rs1799945, rs381815, rs2681492, rs10850411, rs1173771, rs11953630, rs13082711, rs13107325, rs13139571, rs1327235, rs17608766, rs12946454, rs3184504, rs1378942, rs2521501, rs11191548, rs2932538, rs3774372, rs419076, rs4373814, rs7129220, rs805303, rs932764, rs16998073, rs1530440, rs16948048 |
| Yun-Hee Choi | 2014 | Mexican Americans | 2 | chromosome 3 | rs10510257, rs1047115 |
| Yue Qi | 2014 | China/Asians | 9 | GWAS of northeastern  Han Chinese | rs17030613, rs16849225, rs1173766, rs11066280, rs35444, rs880315, rs16998073, rs11191548, rs17249754 |
| Xiangfeng Lu | 2015 | China/Asians | 22 | GWAS of East Asians, identify and replicate 19 in Chinese | rs880315, rs17030613, rs10745332, rs16849225, rs820430, rs9815354, rs9810888, rs1902859, rs6825911, rs13143871, rs1173766, rs1799945, rs9266359, rs2021783, rs4409766, rs4757391, rs17249754, rs11066280, rs1991391, rs35444, rs11067763, rs1887320 |
| Nam-Kyoo Lim | 2015 | Korean/Asians | 4 | GWAS, the KARE project and Health 2 cohort data | rs995322, rs17249754, rs1378942, rs12945290 |
| Teemu J. Niiranen | 2016 | Finland/whites | 32 | GWAS | NR |

SNP: single nucleotide polymorphism; GWAS: Genome Wide Association Study; NR: not report. Rs1378942 was chose in both Sweden and Korean studies; rs17249754 in Korean and 2 Chinese studies; rs11191548 and rs16998073 from Sweden were the same with two Chinese studies; in two Chinese studies, 7 SNPs(rs17030613, rs16849225, rs1173766, rs11066280, rs35444, rs880315 and rs17249754) were the same.
