# Supplementary material for: Recent development of risk-prediction models for incident hypertension: An updated systematic review
Source: PLoS One. 2017 Oct 30;12(10):e0187240. doi: 10.1371/journal.pone.0187240 (PMC5662179; doi:10.1371/journal.pone.0187240)
Supplement: S3 Table — AUC means the area under the receiver operating characteristic curve; CI means confidence interval; JNC—VII definition means the definition of hypertension is based on the Joint National Committee (JNC)—VII definition of hypertension (i.e., SBP/DBP ≥140/90 mmHg or use of antihypertension medications); NR means not reported. First author and year represent study. (DOCX) [file pone.0187240.s004.docx]

S3 Table. External validation of Framingham model

| First author | Year | Country/Ethnicity | Study design | Definition | Follow up (years) | AUC/C-statistic | Calibration |
| --- | --- | --- | --- | --- | --- | --- | --- |
| Mika Kivimäki | 2009 | England/ mainly Whites | prospective | JNC - VII definition | 5 | NR/0.80 | Hosmer–Lemeshow χ^2^=11.5(<20) |
| Paul Muntner | 2010 | Caucasian, African-American,  Hispanic, and Asian - primarily of Chinese descent | prospective | JNC - VII definition | 4-8 | 0.788 (95% CI: 0.773, 0.804)/NR | Hosmer-Lemeshow goodness of fit p<0.001 |
| Henry | 2013 | Northeast Germany/whites | prospective | JNC - VII definition | 5 | 0.77 (95% CI 0.73–0.82)/NR | Hosmer–Lemeshow χ^2^= 11.26(P=0.19) |
| Nam-Kyoo Lim | 2013 | Korean/Asians | prospective | JNC - VII definition | 4 | 0.789 (95% CI, 0.764–0.815)/NR | Hosmer–Lemeshow χ2=29.73 (P=.0002) |
| April P. Carson | 2013 | America/African-American and white | prospective | JNC - VII definition | 25 | 0.84, 95% CI=0.83, 0.85)/NR | Hosmer-Lemeshow χ2= 249.4; p<0.001 |
| Liqiang Zheng | 2014 | China/Chinese | prospective | JNC - VII definition | 4.8 | NR/0.610  (95%CI: 0.602, 0.618) | Hosmer-Lemeshow χ2=8,227.1 (P < 0.0001) |
| Nam-Kyoo Lim | 2016 | Korean/Asians | prospective | JNC - VII definition | 4 | 0.729/NR | Hosmer-Lemeshow goodness of fit p<0.001 |

AUC means the area under the receiver operating characteristic curve; CI means confidence interval;JNC - VII definition means the definition of hypertension is based on the Joint National Committee (JNC) - VII definition of hypertension(i.e., SBP/DBP ≥140/90 mmHg or use of antihypertension medications); NR means not report. First author and year represent study.
