# Supplementary material for: Recent development of risk-prediction models for incident hypertension: An updated systematic review
Source: PLoS One. 2017 Oct 30;12(10):e0187240. doi: 10.1371/journal.pone.0187240 (PMC5662179; doi:10.1371/journal.pone.0187240)
Supplement: S1 File — (DOCX) [file pone.0187240.s005.docx]

**Meta-analysis of 35 prediction models**

Study | ES [95% Conf. Interval] % Weight

---------------------+---------------------------------------------------

Demographic indices | 0.652 0.639 0.665 2.87

Swedish nongenetic r | 0.662 0.653 0.671 2.88

Swedish risk model | 0.664 0.655 0.673 2.88

WHS Simplified Model | 0.703 0.694 0.712 2.88

WHS inclusive risk p | 0.705 0.696 0.714 2.88

WHS Simplified Model | 0.705 0.696 0.714 2.88

Demographic indices | 0.713 0.703 0.723 2.88

China risk predictio | 0.717 0.703 0.731 2.86

China risk predictio | 0.721 0.707 0.735 2.87

TLGS risk score (201 | 0.727 0.714 0.740 2.87

TLGS risk multivaria | 0.731 0.718 0.744 2.87

Taiwan BP clinical r | 0.732 0.714 0.749 2.85

Taiwan BP clinical r | 0.735 0.718 0.752 2.85

TLGS risk multivaria | 0.741 0.728 0.753 2.87

Prediction for women | 0.753 0.741 0.765 2.87

ARIC/CHC risk score | 0.755 0.747 0.763 2.88

biomarker-based risk | 0.755 0.747 0.763 2.88

TLGS risk prediction | 0.760 0.748 0.772 2.87

Prediction for men ( | 0.761 0.751 0.771 2.88

InterASIA risk predi | 0.774 0.765 0.783 2.88

SHIP risk model for | 0.780 0.751 0.808 2.78

Framingham risk scor | 0.788 0.768 0.807 2.84

KoGES risk score (20 | 0.790 0.778 0.801 2.87

SHIP risk model for | 0.790 0.761 0.817 2.79

the average blood pr | 0.794 0.782 0.806 2.87

Whitehall II Repeat | 0.799 0.787 0.811 2.87

Whitehall II risk sc | 0.800 0.788 0.812 2.87

biomarker-based risk | 0.801 0.792 0.810 2.88

rural India risk sco | 0.802 0.755 0.845 2.64

genetic risk predict | 0.803 0.785 0.820 2.85

marginal model (2014 | 0.839 0.803 0.872 2.74

Japanese risk score | 0.858 0.852 0.864 2.89

Japanese risk predic | 0.861 0.855 0.866 2.89

TLGS risk prediction | 0.910 0.902 0.918 2.88

conditional model (2 | 0.973 0.956 0.986 2.86

---------------------+---------------------------------------------------

D+L pooled ES | 0.767 0.742 0.792 100.00

---------------------+---------------------------------------------------

Heterogeneity calculated by formula

Q = SIGMA_i{ (1/variance_i)*(effect_i - effect_pooled)^2 }

where variance_i = ((upper limit - lower limit)/(2*z))^2

Heterogeneity chi-squared = 6325.59 (d.f. = 34) p = 0.000

I-squared (variation in ES attributable to heterogeneity) = 99.5%

Estimate of between-study variance Tau-squared = 0.0055

Test of ES=0 : z= 60.67 p = 0.000

**Tests for Publication Bias**

Begg's Test

adj. Kendall's Score (P-Q) = 38

Std. Dev. of Score = 70.42 (corrected for ties)

Number of Studies = 35

z = 0.54

Pr > |z| = 0.589

z = 0.53 (continuity corrected)

Pr > |z| = 0.599 (continuity corrected)

Egger's test

------------------------------------------------------------------------------

Std_Eff | Coef. Std. Err. t P>|t| [95% Conf. Interval]

-------------+----------------------------------------------------------------

slope | 2.223858 .1547023 14.38 0.000 1.909114 2.538602

bias | -.5018431 1.011743 -0.50 0.623 -2.560249 1.556563

------------------------------------------------------------------------------
